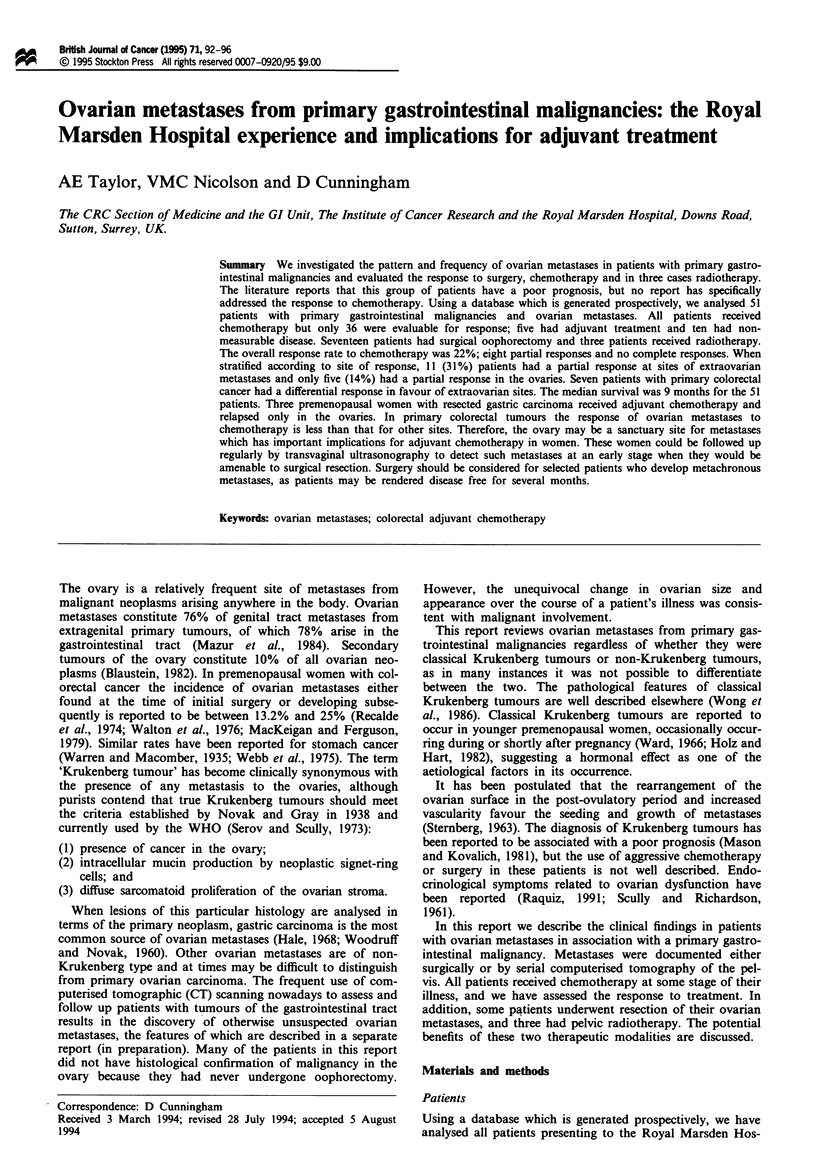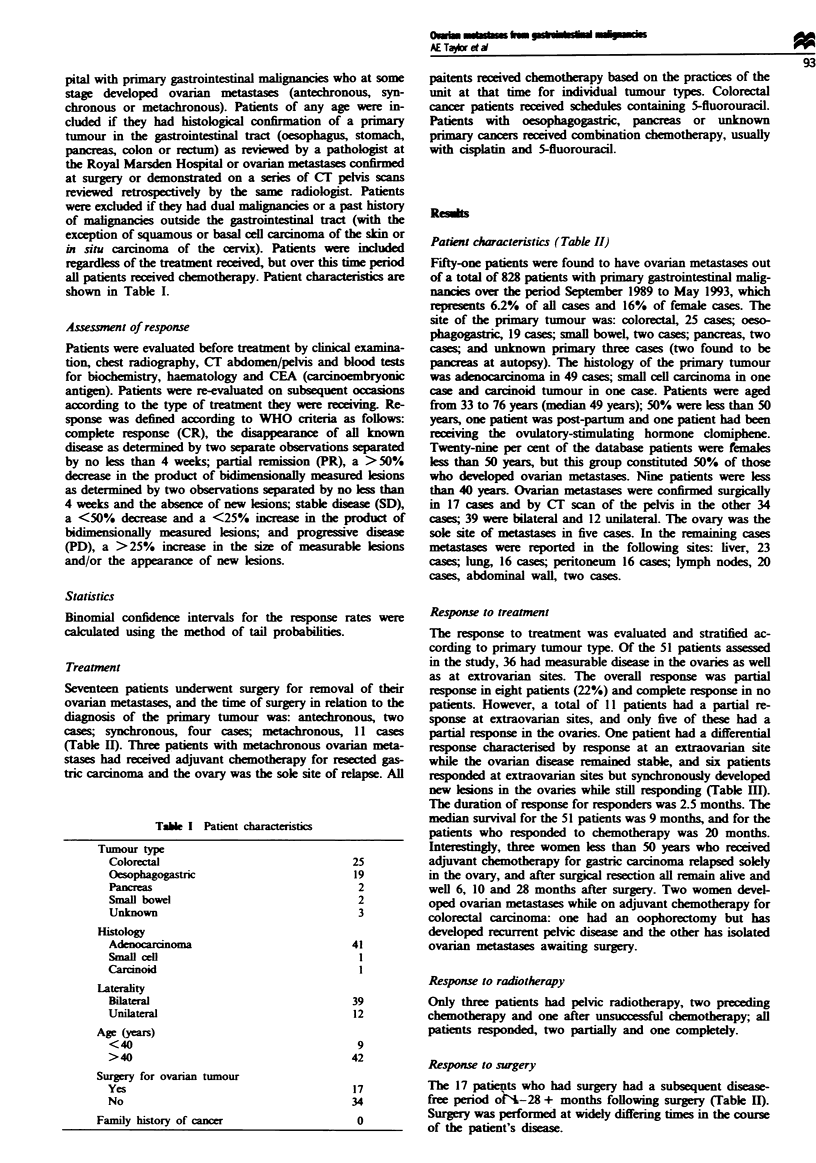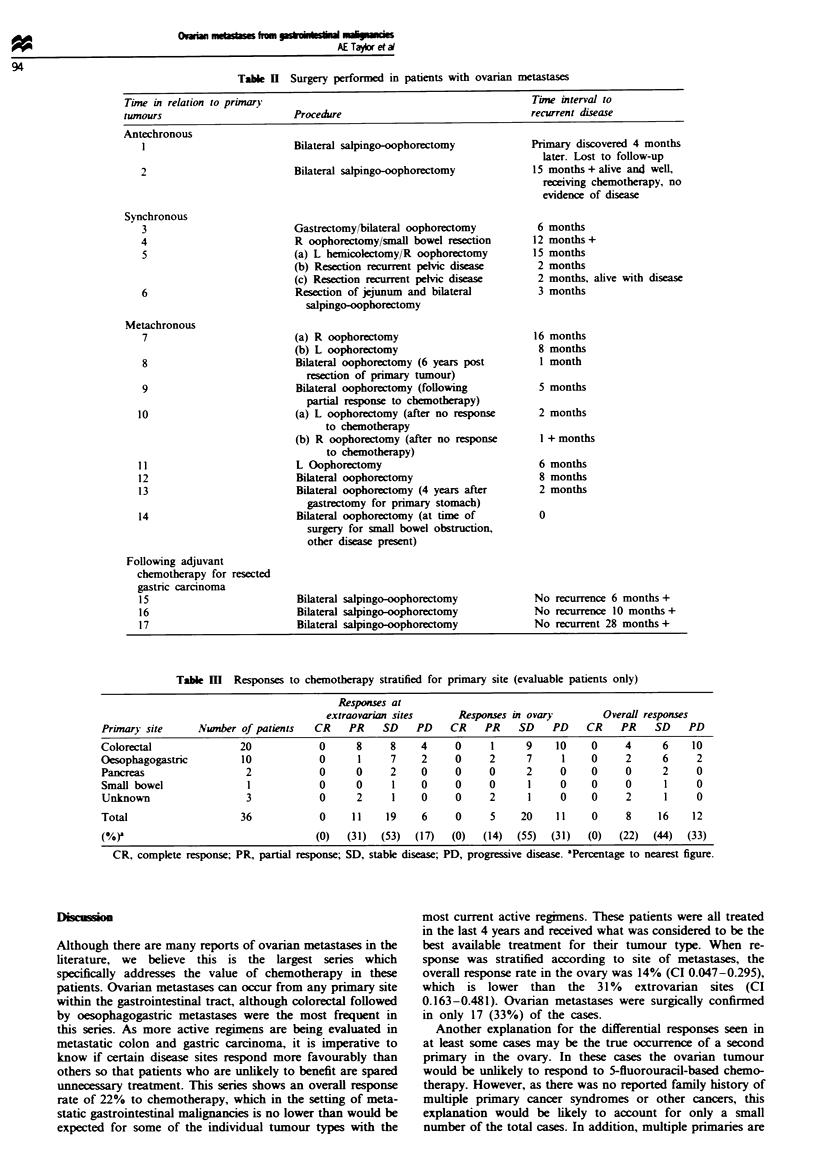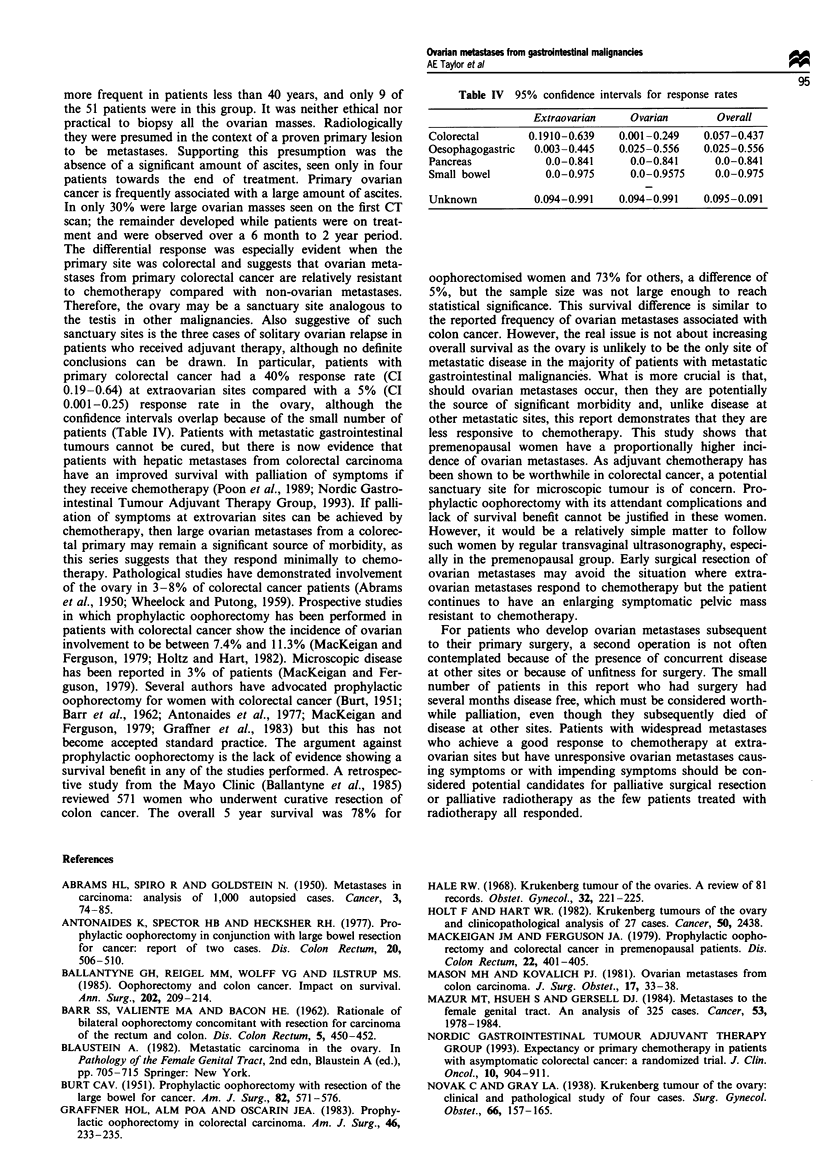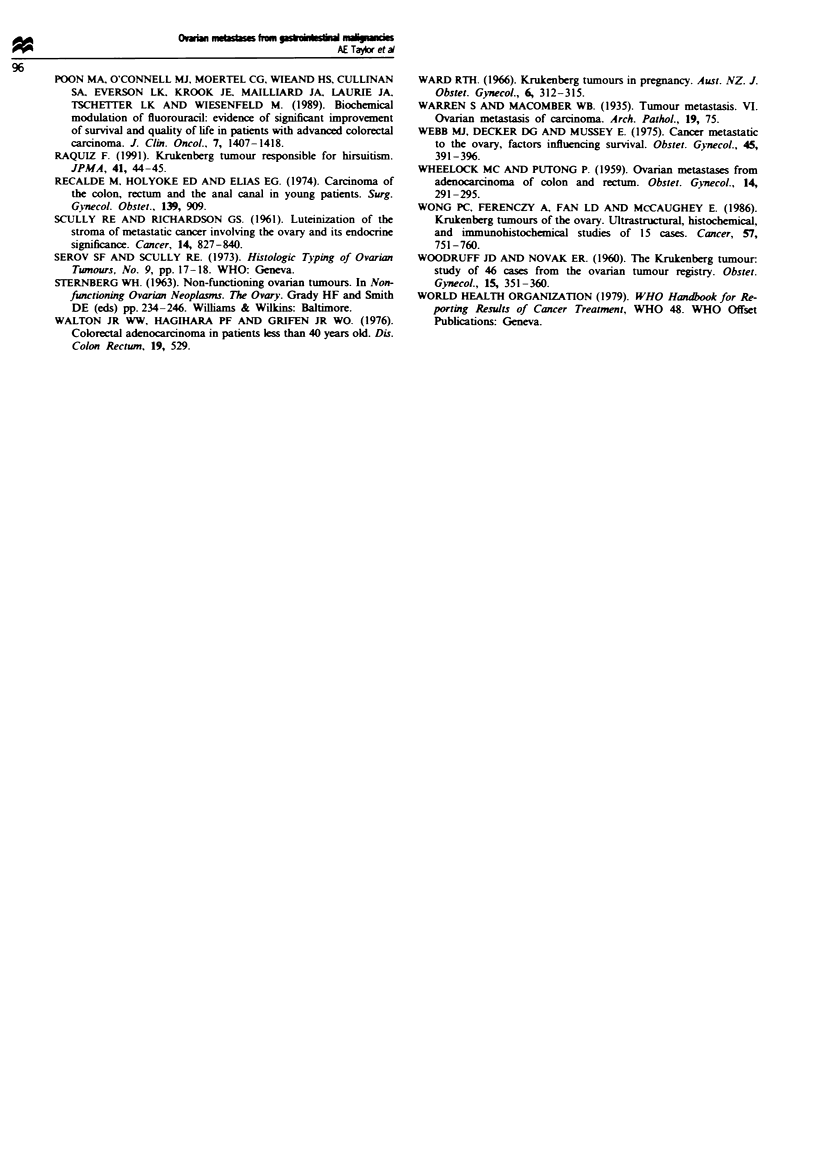# Ovarian metastases from primary gastrointestinal malignancies: the Royal Marsden Hospital experience and implications for adjuvant treatment.

**DOI:** 10.1038/bjc.1995.18

**Published:** 1995-01

**Authors:** A. E. Taylor, V. M. Nicolson, D. Cunningham

**Affiliations:** CRC Section of Medicine, Royal Marsden Hospital, Sutton, Surrey, UK.

## Abstract

We investigated the pattern and frequency of ovarian metastases in patients with primary gastrointestinal malignancies and evaluated the response to surgery, chemotherapy and in three cases radiotherapy. The literature reports that this group of patients have a poor prognosis, but no report has specifically addressed the response to chemotherapy. Using a database which is generated prospectively, we analysed 51 patients with primary gastrointestinal malignancies and ovarian metastases. All patients received chemotherapy but only 36 were evaluable for response; five had adjuvant treatment and ten had non-measurable disease. Seventeen patients had surgical oophorectomy and three patients received radiotherapy. The overall response rate to chemotherapy was 22%; eight partial responses and no complete responses. When stratified according to site of response, 11 (31%) patients had a partial response at sites of extraovarian metastases and only five (14%) had a partial response in the ovaries. Seven patients with primary colorectal cancer had a differential response in favour of extraovarian sites. The median survival was 9 months for the 51 patients. Three premenopausal women with resected gastric carcinoma received adjuvant chemotherapy and relapsed only in the ovaries. In primary colorectal tumours the response of ovarian metastases to chemotherapy is less than that for other sites. Therefore, the ovary may be a sanctuary site for metastases which has important implications for adjuvant chemotherapy in women. These women could be followed up regularly by transvaginal ultrasonography to detect such metastases at an early stage when they would be amenable to surgical resection. Surgery should be considered for selected patients who develop metachronous metastases, as patients may be rendered disease free for several months.


© 1995 Stockton Press All rights reserved 0007-0920/95 $9.00

### Ovarian metastases from primary gastrointestinal malignancies: the Royal Marsden Hospital experience and implications for adjuvant treatment

AE Taylor, VMC Nicolson and D Cunningham

*The CRC Section of Medicine and the GI Unit, The Institute of Cancer Research and the Royal Marsden Hospital, Downs Road, Sutton, Surrey, UK.*

**Summary** We investigated the pattern and frequency of ovarian metastases in patients with primary gastrointestinal malignancies and evaluated the response to surgery, chemotherapy and in three cases radiotherapy. The literature reports that this group of patients have a poor prognosis, but no report has specifically addressed the response to chemotherapy. Using a database which is generated prospectively, we analysed 51 patients with primary gastrointestinal malignancies and ovarian metastases. All patients received chemotherapy but only 36 were evaluable for response; five had adjuvant treatment and ten had non-measurable disease. Seventeen patients had surgical oophorectomy and three patients received radiotherapy. The overall response rate to chemotherapy was 22%; eight partial responses and no complete responses. When stratified according to site of response, 11 (31%) patients had a partial response at sites of extraovarian metastases and only five (14%) had a partial response in the ovaries. Seven patients with primary colorectal cancer had a differential response in favour of extraovarian sites. The median survival was 9 months for the 51 patients. Three premenopausal women with resected gastric carcinoma received adjuvant chemotherapy and relapsed only in the ovaries. In primary colorectal tumours the response of ovarian metastases to chemotherapy is less than that for other sites. Therefore, the ovary may be a sanctuary site for metastases which has important implications for adjuvant chemotherapy in women. These women could be followed up regularly by transvaginal ultrasonography to detect such metastases at an early stage when they would be amenable to surgical resection. Surgery should be considered for selected patients who develop metachronous metastases, as patients may be rendered disease free for several months.

**Keywords:** ovarian metastases; colorectal adjuvant chemotherapy

The ovary is a relatively frequent site of metastases from malignant neoplasms arising anywhere in the body. Ovarian metastases constitute 76% of genital tract metastases from extragenital primary tumours, of which 78% arise in the gastrointestinal tract (Mazur *et al.*, 1984). Secondary tumours of the ovary constitute 10% of all ovarian neoplasms (Blaustein, 1982). In premenopausal women with colorectal cancer the incidence of ovarian metastases either found at the time of initial surgery or developing subsequently is reported to be between 13.2% and 25% (Recalde *et al.*, 1974; Walton *et al.*, 1976; MacKeigan and Ferguson, 1979). Similar rates have been reported for stomach cancer (Warren and Macomber, 1935; Webb *et al.*, 1975). The term 'Krukenberg tumour' has become clinically synonymous with the presence of any metastasis to the ovaries, although purists contend that true Krukenberg tumours should meet the criteria established by Novak and Gray in 1938 and currently used by the WHO (Serov and Scully, 1973):

(1) presence of cancer in the ovary;
(2) intracellular mucin production by neoplastic signet-ring cells; and
(3) diffuse sarcomatoid proliferation of the ovarian stroma.

When lesions of this particular histology are analysed in terms of the primary neoplasm, gastric carcinoma is the most common source of ovarian metastases (Hale, 1968; Woodruff and Novak, 1960). Other ovarian metastases are of non-Krukenberg type and at times may be difficult to distinguish from primary ovarian carcinoma. The frequent use of computerised tomographic (CT) scanning nowadays to assess and follow up patients with tumours of the gastrointestinal tract results in the discovery of otherwise unsuspected ovarian metastases, the features of which are described in a separate report (in preparation). Many of the patients in this report did not have histological confirmation of malignancy in the ovary because they had never undergone oophorectomy. However, the unequivocal change in ovarian size and appearance over the course of a patient's illness was consistent with malignant involvement.

This report reviews ovarian metastases from primary gastrointestinal malignancies regardless of whether they were classical Krukenberg tumours or non-Krukenberg tumours, as in many instances it was not possible to differentiate between the two. The pathological features of classical Krukenberg tumours are well described elsewhere (Wong *et al.*, 1986). Classical Krukenberg tumours are reported to occur in younger premenopausal women, occasionally occurring during or shortly after pregnancy (Ward, 1966; Holz and Hart, 1982), suggesting a hormonal effect as one of the aetiological factors in its occurrence.

It has been postulated that the rearrangement of the ovarian surface in the post-ovulatory period and increased vascularity favour the seeding and growth of metastases (Sternberg, 1963). The diagnosis of Krukenberg tumours has been reported to be associated with a poor prognosis (Mason and Kovalich, 1981), but the use of aggressive chemotherapy or surgery in these patients is not well described. Endocrinological symptoms related to ovarian dysfunction have been reported (Raquiz, 1991; Scully and Richardson, 1961).

In this report we describe the clinical findings in patients with ovarian metastases in association with a primary gastrointestinal malignancy. Metastases were documented either surgically or by serial computerised tomography of the pelvis. All patients received chemotherapy at some stage of their illness, and we have assessed the response to treatment. In addition, some patients underwent resection of their ovarian metastases, and three had pelvic radiotherapy. The potential benefits of these two therapeutic modalities are discussed.

### Materials and methods

### *Patients*

Using a database which is generated prospectively, we have analysed all patients presenting to the Royal Marsden Hos-

Correspondence: D Cunningham
Received 3 March 1994; revised 28 July 1994; accepted 5 August 1994

pital with primary gastrointestinal malignancies who at some stage developed ovarian metastases (antechronous, synchronous or metachronous). Patients of any age were included if they had histological confirmation of a primary tumour in the gastrointestinal tract (oesophagus, stomach, pancreas, colon or rectum) as reviewed by a pathologist at the Royal Marsden Hospital or ovarian metastases confirmed at surgery or demonstrated on a series of CT pelvis scans reviewed retrospectively by the same radiologist. Patients were excluded if they had dual malignancies or a past history of malignancies outside the gastrointestinal tract (with the exception of squamous or basal cell carcinoma of the skin or *in situ* carcinoma of the cervix). Patients were included regardless of the treatment received, but over this time period all patients received chemotherapy. Patient characteristics are shown in Table I.

*Assessment of response*

Patients were evaluated before treatment by clinical examination, chest radiography, CT abdomen/pelvis and blood tests for biochemistry, haematology and CEA (carcinoembryonic antigen). Patients were re-evaluated on subsequent occasions according to the type of treatment they were receiving. Response was defined according to WHO criteria as follows: complete response (CR), the disappearance of all known disease as determined by two separate observations separated by no less than 4 weeks; partial remission (PR), a >50% decrease in the product of bidimensionally measured lesions as determined by two observations separated by no less than 4 weeks and the absence of new lesions; stable disease (SD), a <50% decrease and a <25% increase in the product of bidimensionally measured lesions; and progressive disease (PD), a >25% increase in the size of measurable lesions and/or the appearance of new lesions.

*Statistics*

Binomial confidence intervals for the response rates were calculated using the method of tail probabilities.

*Treatment*

Seventeen patients underwent surgery for removal of their ovarian metastases, and the time of surgery in relation to the diagnosis of the primary tumour was: antechronous, two cases; synchronous, four cases; metachronous, 11 cases (Table II). Three patients with metachronous ovarian metastases had received adjuvant chemotherapy for resected gastric carcinoma and the ovary was the sole site of relapse. All paitents received chemotherapy based on the practices of the unit at that time for individual tumour types. Colorectal cancer patients received schedules containing 5-fluorouracil. Patients with oesophagogastric, pancreas or unknown primary cancers received combination chemotherapy, usually with cisplatin and 5-fluorouracil.

**Table I** Patient characteristics

| | |
|---|---|
| Tumour type | |
| Colorectal | 25 |
| Oesophagogastric | 19 |
| Pancreas | 2 |
| Small bowel | 2 |
| Unknown | 3 |
| Histology | |
| Adenocarcinoma | 41 |
| Small cell | 1 |
| Carcinoid | 1 |
| Laterality | |
| Bilateral | 39 |
| Unilateral | 12 |
| Age (years) | |
| <40 | 9 |
| >40 | 42 |
| Surgery for ovarian tumour | |
| Yes | 17 |
| No | 34 |
| Family history of cancer | 0 |

### Results

*Patient characteristics (Table II)*

Fifty-one patients were found to have ovarian metastases out of a total of 828 patients with primary gastrointestinal malignancies over the period September 1989 to May 1993, which represents 6.2% of all cases and 16% of female cases. The site of the primary tumour was: colorectal, 25 cases; oesophagogastric, 19 cases; small bowel, two cases; pancreas, two cases; and unknown primary three cases (two found to be pancreas at autopsy). The histology of the primary tumour was adenocarcinoma in 49 cases; small cell carcinoma in one case and carcinoid tumour in one case. Patients were aged from 33 to 76 years (median 49 years); 50% were less than 50 years, one patient was post-partum and one patient had been receiving the ovulatory-stimulating hormone clomiphene. Twenty-nine per cent of the database patients were females less than 50 years, but this group constituted 50% of those who developed ovarian metastases. Nine patients were less than 40 years. Ovarian metastases were confirmed surgically in 17 cases and by CT scan of the pelvis in the other 34 cases; 39 were bilateral and 12 unilateral. The ovary was the sole site of metastases in five cases. In the remaining cases metastases were reported in the following sites: liver, 23 cases; lung, 16 cases; peritoneum 16 cases; lymph nodes, 20 cases, abdominal wall, two cases.

*Response to treatment*

The response to treatment was evaluated and stratified according to primary tumour type. Of the 51 patients assessed in the study, 36 had measurable disease in the ovaries as well as at extrovarian sites. The overall response was partial response in eight patients (22%) and complete response in no patients. However, a total of 11 patients had a partial response at extraovarian sites, and only five of these had a partial response in the ovaries. One patient had a differential response characterised by response at an extraovarian site while the ovarian disease remained stable, and six patients responded at extraovarian sites but synchronously developed new lesions in the ovaries while still responding (Table III). The duration of response for responders was 2.5 months. The median survival for the 51 patients was 9 months, and for the patients who responded to chemotherapy was 20 months. Interestingly, three women less than 50 years who received adjuvant chemotherapy for gastric carcinoma relapsed solely in the ovary, and after surgical resection all remain alive and well 6, 10 and 28 months after surgery. Two women developed ovarian metastases while on adjuvant chemotherapy for colorectal carcinoma: one had an oophorectomy but has developed recurrent pelvic disease and the other has isolated ovarian metastases awaiting surgery.

*Response to radiotherapy*

Only three patients had pelvic radiotherapy, two preceding chemotherapy and one after unsuccessful chemotherapy; all patients responded, two partially and one completely.

*Response to surgery*

The 17 patients who had surgery had a subsequent disease-free period of [illegible]–28+ months following surgery (Table II). Surgery was performed at widely differing times in the course of the patient's disease.

Table II Surgery performed in patients with ovarian metastases

| Time in relation to primary tumours | Procedure | Time interval to recurrent disease |
|---|---|---|
| Antechronous | | |
| 1 | Bilateral salpingo-oophorectomy | Primary discovered 4 months later. Lost to follow-up |
| 2 | Bilateral salpingo-oophorectomy | 15 months + alive and well, receiving chemotherapy, no evidence of disease |
| Synchronous | | |
| 3 | Gastrectomy/bilateral oophorectomy | 6 months |
| 4 | R oophorectomy/small bowel resection | 12 months + |
| 5 | (a) L hemicolectomy/R oophorectomy | 15 months |
| | (b) Resection recurrent pelvic disease | 2 months |
| | (c) Resection recurrent pelvic disease | 2 months, alive with disease |
| 6 | Resection of jejunum and bilateral salpingo-oophorectomy | 3 months |
| Metachronous | | |
| 7 | (a) R oophorectomy | 16 months |
| | (b) L oophorectomy | 8 months |
| 8 | Bilateral oophorectomy (6 years post resection of primary tumour) | 1 month |
| 9 | Bilateral oophorectomy (following partial response to chemotherapy) | 5 months |
| 10 | (a) L oophorectomy (after no response to chemotherapy | 2 months |
| | (b) R oophorectomy (after no response to chemotherapy) | 1 + months |
| 11 | L Oophorectomy | 6 months |
| 12 | Bilateral oophorectomy | 8 months |
| 13 | Bilateral oophorectomy (4 years after gastrectomy for primary stomach) | 2 months |
| 14 | Bilateral oophorectomy (at time of surgery for small bowel obstruction, other disease present) | 0 |
| Following adjuvant chemotherapy for resected gastric carcinoma | | |
| 15 | Bilateral salpingo-oophorectomy | No recurrence 6 months + |
| 16 | Bilateral salpingo-oophorectomy | No recurrence 10 months + |
| 17 | Bilateral salpingo-oophorectomy | No recurrent 28 months + |

Table III Responses to chemotherapy stratified for primary site (evaluable patients only)

| | | Responses at extraovarian sites | | | | Responses in ovary | | | | Overall responses | | | |
|---|---|---|---|---|---|---|---|---|---|---|---|---|---|
| Primary site | Number of patients | CR | PR | SD | PD | CR | PR | SD | PD | CR | PR | SD | PD |
| Colorectal | 20 | 0 | 8 | 8 | 4 | 0 | 1 | 9 | 10 | 0 | 4 | 6 | 10 |
| Oesophagogastric | 10 | 0 | 1 | 7 | 2 | 0 | 2 | 7 | 1 | 0 | 2 | 6 | 2 |
| Pancreas | 2 | 0 | 0 | 2 | 0 | 0 | 0 | 2 | 0 | 0 | 0 | 2 | 0 |
| Small bowel | 1 | 0 | 0 | 1 | 0 | 0 | 0 | 1 | 0 | 0 | 0 | 1 | 0 |
| Unknown | 3 | 0 | 2 | 1 | 0 | 0 | 2 | 1 | 0 | 0 | 2 | 1 | 0 |
| Total | 36 | 0 | 11 | 19 | 6 | 0 | 5 | 20 | 11 | 0 | 8 | 16 | 12 |
| (%)[a] | | (0) | (31) | (53) | (17) | (0) | (14) | (55) | (31) | (0) | (22) | (44) | (33) |

CR, complete response; PR, partial response; SD, stable disease; PD, progressive disease. [a]Percentage to nearest figure.

### Discussion

Although there are many reports of ovarian metastases in the literature, we believe this is the largest series which specifically addresses the value of chemotherapy in these patients. Ovarian metastases can occur from any primary site within the gastrointestinal tract, although colorectal followed by oesophagogastric metastases were the most frequent in this series. As more active regimens are being evaluated in metastatic colon and gastric carcinoma, it is imperative to know if certain disease sites respond more favourably than others so that patients who are unlikely to benefit are spared unnecessary treatment. This series shows an overall response rate of 22% to chemotherapy, which in the setting of metastatic gastrointestinal malignancies is no lower than would be expected for some of the individual tumour types with the most current active regimens. These patients were all treated in the last 4 years and received what was considered to be the best available treatment for their tumour type. When response was stratified according to site of metastases, the overall response rate in the ovary was 14% (CI 0.047–0.295), which is lower than the 31% extrovarian sites (CI 0.163–0.481). Ovarian metastases were surgically confirmed in only 17 (33%) of the cases.

Another explanation for the differential responses seen in at least some cases may be the true occurrence of a second primary in the ovary. In these cases the ovarian tumour would be unlikely to respond to 5-fluorouracil-based chemotherapy. However, as there was no reported family history of multiple primary cancer syndromes or other cancers, this explanation would be likely to account for only a small number of the total cases. In addition, multiple primaries are more frequent in patients less than 40 years, and only 9 of the 51 patients were in this group. It was neither ethical nor practical to biopsy all the ovarian masses. Radiologically they were presumed in the context of a proven primary lesion to be metastases. Supporting this presumption was the absence of a significant amount of ascites, seen only in four patients towards the end of treatment. Primary ovarian cancer is frequently associated with a large amount of ascites. In only 30% were large ovarian masses seen on the first CT scan; the remainder developed while patients were on treatment and were observed over a 6 month to 2 year period. The differential response was especially evident when the primary site was colorectal and suggests that ovarian metastases from primary colorectal cancer are relatively resistant to chemotherapy compared with non-ovarian metastases. Therefore, the ovary may be a sanctuary site analogous to the testis in other malignancies. Also suggestive of such sanctuary sites is the three cases of solitary ovarian relapse in patients who received adjuvant therapy, although no definite conclusions can be drawn. In particular, patients with primary colorectal cancer had a 40% response rate (CI 0.19–0.64) at extraovarian sites compared with a 5% (CI 0.001–0.25) response rate in the ovary, although the confidence intervals overlap because of the small number of patients (Table IV). Patients with metastatic gastrointestinal tumours cannot be cured, but there is now evidence that patients with hepatic metastases from colorectal carcinoma have an improved survival with palliation of symptoms if they receive chemotherapy (Poon *et al.*, 1989; Nordic Gastrointestinal Tumour Adjuvant Therapy Group, 1993). If palliation of symptoms at extrovarian sites can be achieved by chemotherapy, then large ovarian metastases from a colorectal primary may remain a significant source of morbidity, as this series suggests that they respond minimally to chemotherapy. Pathological studies have demonstrated involvement of the ovary in 3–8% of colorectal cancer patients (Abrams *et al.*, 1950; Wheelock and Putong, 1959). Prospective studies in which prophylactic oophorectomy has been performed in patients with colorectal cancer show the incidence of ovarian involvement to be between 7.4% and 11.3% (MacKeigan and Ferguson, 1979; Holtz and Hart, 1982). Microscopic disease has been reported in 3% of patients (MacKeigan and Ferguson, 1979). Several authors have advocated prophylactic oophorectomy for women with colorectal cancer (Burt, 1951; Barr *et al.*, 1962; Antonaides *et al.*, 1977; MacKeigan and Ferguson, 1979; Graffner *et al.*, 1983) but this has not become accepted standard practice. The argument against prophylactic oophorectomy is the lack of evidence showing a survival benefit in any of the studies performed. A retrospective study from the Mayo Clinic (Ballantyne *et al.*, 1985) reviewed 571 women who underwent curative resection of colon cancer. The overall 5 year survival was 78% for oophorectomised women and 73% for others, a difference of 5%, but the sample size was not large enough to reach statistical significance. This survival difference is similar to the reported frequency of ovarian metastases associated with colon cancer. However, the real issue is not about increasing overall survival as the ovary is unlikely to be the only site of metastatic disease in the majority of patients with metastatic gastrointestinal malignancies. What is more crucial is that, should ovarian metastases occur, then they are potentially the source of significant morbidity and, unlike disease at other metastatic sites, this report demonstrates that they are less responsive to chemotherapy. This study shows that premenopausal women have a proportionally higher incidence of ovarian metastases. As adjuvant chemotherapy has been shown to be worthwhile in colorectal cancer, a potential sanctuary site for microscopic tumour is of concern. Prophylactic oophorectomy with its attendant complications and lack of survival benefit cannot be justified in these women. However, it would be a relatively simple matter to follow such women by regular transvaginal ultrasonography, especially in the premenopausal group. Early surgical resection of ovarian metastases may avoid the situation where extra-ovarian metastases respond to chemotherapy but the patient continues to have an enlarging symptomatic pelvic mass resistant to chemotherapy.

**Table IV** 95% confidence intervals for response rates

| | *Extraovarian* | *Ovarian* | *Overall* |
|---|---|---|---|
| Colorectal | 0.1910–0.639 | 0.001–0.249 | 0.057–0.437 |
| Oesophagogastric | 0.003–0.445 | 0.025–0.556 | 0.025–0.556 |
| Pancreas | 0.0–0.841 | 0.0–0.841 | 0.0–0.841 |
| Small bowel | 0.0–0.975 | 0.0–0.9575 | 0.0–0.975 |
| | | – | |
| Unknown | 0.094–0.991 | 0.094–0.991 | 0.095–0.091 |

For patients who develop ovarian metastases subsequent to their primary surgery, a second operation is not often contemplated because of the presence of concurrent disease at other sites or because of unfitness for surgery. The small number of patients in this report who had surgery had several months disease free, which must be considered worthwhile palliation, even though they subsequently died of disease at other sites. Patients with widespread metastases who achieve a good response to chemotherapy at extra-ovarian sites but have unresponsive ovarian metastases causing symptoms or with impending symptoms should be considered potential candidates for palliative surgical resection or palliative radiotherapy as the few patients treated with radiotherapy all responded.

### References

ABRAMS HL, SPIRO R AND GOLDSTEIN N. (1950). Metastases in carcinoma: analysis of 1,000 autopsied cases. *Cancer*, **3**, 74–85.

ANTONAIDES K, SPECTOR HB AND HECKSHER RH. (1977). Prophylactic oophorectomy in conjunction with large bowel resection for cancer: report of two cases. *Dis. Colon Rectum*, **20**, 506–510.

BALLANTYNE GH, REIGEL MM, WOLFF VG AND ILSTRUP MS. (1985). Oophorectomy and colon cancer. Impact on survival. *Ann. Surg.*, **202**, 209–214.

BARR SS, VALIENTE MA AND BACON HE. (1962). Rationale of bilateral oophorectomy concomitant with resection for carcinoma of the rectum and colon. *Dis. Colon Rectum*, **5**, 450–452.

BLAUSTEIN A. (1982). Metastatic carcinoma in the ovary. In *Pathology of the Female Genital Tract*, 2nd edn, Blaustein A (ed.), pp. 705–715 Springer: New York.

BURT CAV. (1951). Prophylactic oophorectomy with resection of the large bowel for cancer. *Am. J. Surg.*, **82**, 571–576.

GRAFFNER HOL, ALM POA AND OSCARIN JEA. (1983). Prophylactic oophorectomy in colorectal carcinoma. *Am. J. Surg.*, **46**, 233–235.

HALE RW. (1968). Krukenberg tumour of the ovaries. A review of 81 records. *Obstet. Gynecol.*, **32**, 221–225.

HOLT F AND HART WR. (1982). Krukenberg tumours of the ovary and clinicopathological analysis of 27 cases. *Cancer*, **50**, 2438.

MACKEIGAN JM AND FERGUSON JA. (1979). Prophylactic oophorectomy and colorectal cancer in premenopausal patients. *Dis. Colon Rectum*, **22**, 401–405.

MASON MH AND KOVALICH PJ. (1981). Ovarian metastases from colon carcinoma. *J. Surg. Obstet.*, **17**, 33–38.

MAZUR MT, HSUEH S AND GERSELL DJ. (1984). Metastases to the female genital tract. An analysis of 325 cases. *Cancer*, **53**, 1978–1984.

NORDIC GASTROINTESTINAL TUMOUR ADJUVANT THERAPY GROUP (1993). Expectancy or primary chemotherapy in patients with asymptomatic colorectal cancer: a randomized trial. *J. Clin. Oncol.*, **10**, 904–911.

NOVAK C AND GRAY LA. (1938). Krukenberg tumour of the ovary: clinical and pathological study of four cases. *Surg. Gynecol. Obstet.*, **66**, 157–165.

POON MA, O'CONNELL MJ, MOERTEL CG, WIEAND HS, CULLINAN SA, EVERSON LK, KROOK JE, MAILLIARD JA, LAURIE JA, TSCHETTER LK AND WIESENFELD M. (1989). Biochemical modulation of fluorouracil: evidence of significant improvement of survival and quality of life in patients with advanced colorectal carcinoma. *J. Clin. Oncol.*, **7**, 1407–1418.

RAQUIZ F. (1991). Krukenberg tumour responsible for hirsuitism. *JPMA*, **41**, 44–45.

RECALDE M, HOLYOKE ED AND ELIAS EG. (1974). Carcinoma of the colon, rectum and the anal canal in young patients. *Surg. Gynecol. Obstet.*, **139**, 909.

SCULLY RE AND RICHARDSON GS. (1961). Luteinization of the stroma of metastatic cancer involving the ovary and its endocrine significance. *Cancer*, **14**, 827–840.

SEROV SF AND SCULLY RE. (1973). *Histologic Typing of Ovarian Tumours, No. 9*, pp. 17–18. WHO: Geneva.

STERNBERG WH. (1963). Non-functioning ovarian tumours. In *Non-functioning Ovarian Neoplasms. The Ovary*. Grady HF and Smith DE (eds) pp. 234–246. Williams & Wilkins: Baltimore.

WALTON JR WW, HAGIHARA PF AND GRIFEN JR WO. (1976). Colorectal adenocarcinoma in patients less than 40 years old. *Dis. Colon Rectum*, **19**, 529.

WARD RTH. (1966). Krukenberg tumours in pregnancy. *Aust. NZ. J. Obstet. Gynecol.*, **6**, 312–315.

WARREN S AND MACOMBER WB. (1935). Tumour metastasis. VI. Ovarian metastasis of carcinoma. *Arch. Pathol.*, **19**, 75.

WEBB MJ, DECKER DG AND MUSSEY E. (1975). Cancer metastatic to the ovary, factors influencing survival. *Obstet. Gynecol.*, **45**, 391–396.

WHEELOCK MC AND PUTONG P. (1959). Ovarian metastases from adenocarcinoma of colon and rectum. *Obstet. Gynecol.*, **14**, 291–295.

WONG PC, FERENCZY A, FAN LD AND McCAUGHEY E. (1986). Krukenberg tumours of the ovary. Ultrastructural, histochemical, and immunohistochemical studies of 15 cases. *Cancer*, **57**, 751–760.

WOODRUFF JD AND NOVAK ER. (1960). The Krukenberg tumour: study of 46 cases from the ovarian tumour registry. *Obstet. Gynecol.*, **15**, 351–360.

WORLD HEALTH ORGANIZATION (1979). *WHO Handbook for Reporting Results of Cancer Treatment*, WHO 48. WHO Offset Publications: Geneva.